# Hypoxia-adenosine axis as therapeutic targets for acute respiratory distress syndrome

**DOI:** 10.3389/fimmu.2024.1328565

**Published:** 2024-01-19

**Authors:** Katherine Figarella, Jieun Kim, Wei Ruan, Tingting Mills, Holger Klaus Eltzschig, Xiaoyi Yuan

**Affiliations:** ^1^Department of Anesthesiology, Critical Care and Pain Medicine, University of Texas Health Science Center at Houston, Houston, TX, United States; ^2^Department of Anesthesiology, The Second Xiangya Hospital, Central South University, Changsha, Hunan, China; ^3^Department of Biochemistry and Molecular Biology, University of Texas Health Science Center at Houston, Houston, TX, United States

**Keywords:** hypoxia, adenosine, ARDS, inflammation, lung injury

## Abstract

The human respiratory and circulatory systems collaborate intricately to ensure oxygen delivery to all cells, which is vital for ATP production and maintaining physiological functions and structures. During limited oxygen availability, hypoxia-inducible factors (HIFs) are stabilized and play a fundamental role in maintaining cellular processes for hypoxia adaptation. First discovered during investigations of erythropoietin production regulation, HIFs influence physiological and pathological processes, including development, inflammation, wound healing, and cancer. HIFs promote extracellular adenosine signaling by enhancing adenosine generation and receptor signaling, representing an endogenous feedback mechanism that curbs excessive inflammation, supports injury resolution, and enhances hypoxia tolerance. This is especially important for conditions that involve tissue hypoxia, such as acute respiratory distress syndrome (ARDS), which globally poses significant health challenges without specific treatment options. Consequently, pharmacological strategies to amplify HIF-mediated adenosine production and receptor signaling are of great importance.

## Introduction

**Figure 1:**
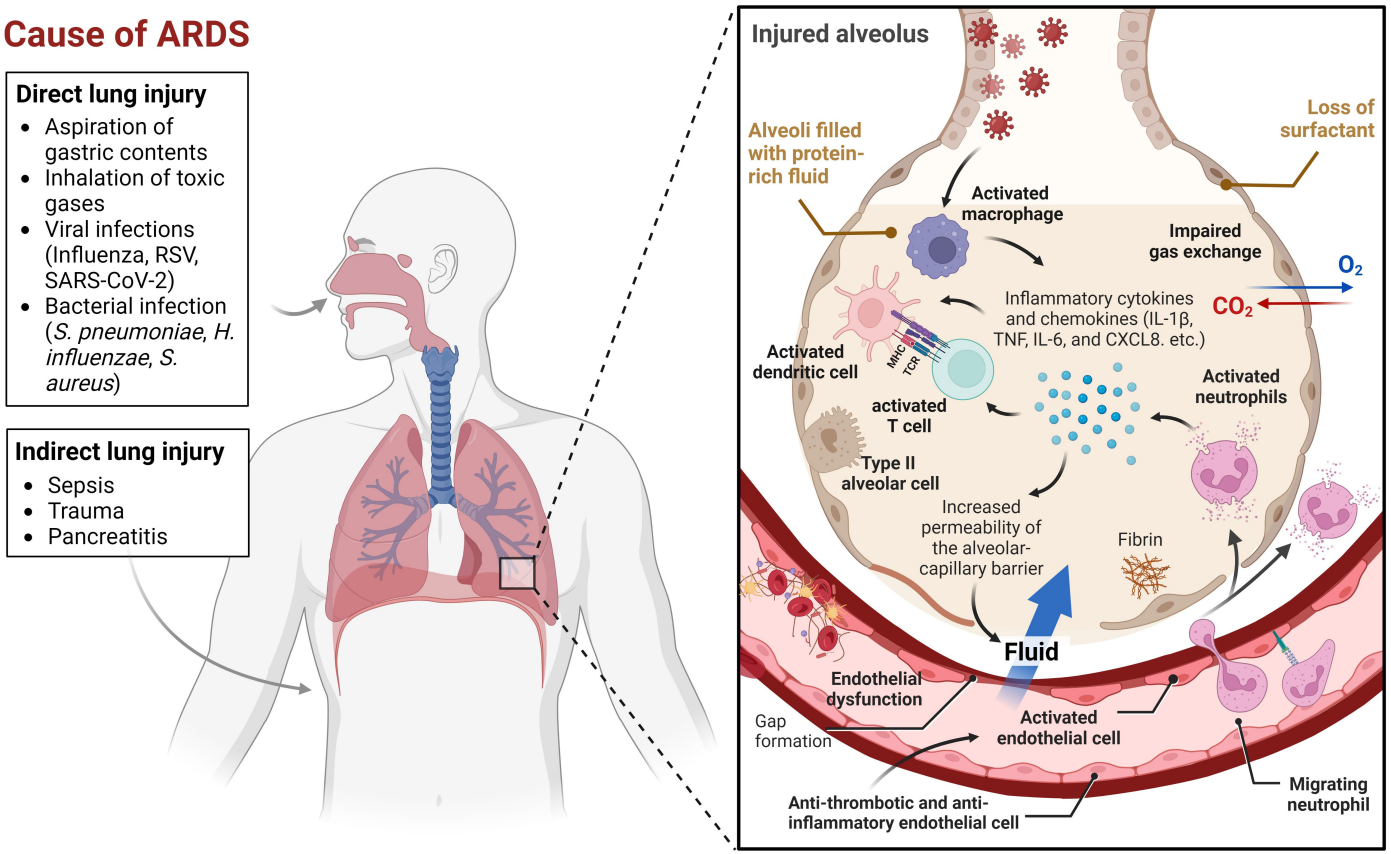
Multifactorial events leading to Acute Respiratory Distress Syndrome (ARDS). This figure illustrates the many factors that can contribute to ARDS. Lung injury can be caused by a variety of direct or indirect insults, or a combination of both. When an initial insult occurs, neutrophils and macrophages are activated, leading to the release of proinflammatory cytokines and chemokines. Some of the key chemokines and cytokines implicated in ARDS include interleukin-1β (IL-1β), tumor necrosis factor (TNF), interleukin-6 (IL-6), and CXCL8 (3). These molecules play a crucial role in the recruitment and activation of immune cells, such as neutrophils, macrophages, dendritic cells, and T cells, which contribute to the inflammatory response in ARDS. Endothelial cells (ECs) coordinate the immune and hemostatic response by shifting from their normal anti-thrombotic and anti-inflammatory phenotype to an activated state of endothelial dysfunction. This shift is a critical host response to infection and injury and is involved in ARDS pathogenesis. The inflammatory response increases the permeability of the alveolar-capillary barrier, which causes protein-rich fluid to leak into the alveolar space, resulting in impaired gas exchange. Meanwhile, abnormalities in surfactant function further exacerbate the disruption of gas exchange. This combination of factors, including inflammation, impaired surfactant function, and increased alveolar-capillary barrier permeability, leads to a progressive decline in oxygenation and respiratory function. The visual representation of this complex process was created using BioRender.com.

Acute lung injury caused by various insults, including pneumonia, sepsis, and trauma, can aggravate and lead to a more severe condition known as acute respiratory distress syndrome (ARDS). The acute onset of respiratory insufficiency, bilateral lung infiltrates on chest imaging, and severe progressive hypoxemia (low oxygen levels in the blood) are key characteristics of ARDS (1). Its pathophysiology is complex and multifactorial, involving direct and indirect lung injury (2). Direct lung injury can be caused by factors such as aspiration of gastric contents, inhalation of toxic gases, or viral and bacterial infections. Indirect lung injury can be caused by systemic factors such as sepsis, trauma, or pancreatitis, which can generate an inflammatory response that ultimately affects the lung (**Figure 1**). The initial insult to the lung induces the activation of neutrophils and macrophages, which release proinflammatory cytokines and chemokines (3). This leads to increased permeability of the alveolar-capillary barrier, allowing protein-rich fluid to leak into the alveolar space and impairing gas exchange. The initial inflammatory response is accompanied by abnormalities in surfactant function, which can further impair gas exchange. Surfactant is a mixture of lipids and proteins that lines the alveoli and helps maintain alveolar stability and prevent collapse (4). The combination of inflammation, impaired surfactant function, and increased alveolar-capillary barrier permeability causes a progressive decline in oxygenation and respiratory function.

Treatment for ARDS primarily focuses on supporting the patient while the lungs heal and addressing the underlying cause of the condition. It includes measures such as mechanical ventilation with low tidal volumes, prone positioning, and administration of supplemental oxygen (5). In some cases, extracorporeal membrane oxygenation (ECMO) may temporarily support oxygenation (5). The therapeutic interventions used to manage the disease are summarized in **Table 1**. Although there have been improvements in providing support and care, the mortality rate of ARDS is still high, ~40% (12). There is still no targeted treatment for ARDS. Thus, a better understanding of the pathophysiology of the complex multifactorial ARDS may lead to the development of novel therapeutic strategies to improve outcomes for patients with this devastating condition.

**Table 1:** Therapeutical interventions applied to patients with ARDS.

Intervention	Aim	Remarks
**Oxygen therapy**	Oxygen is delivered to patients through a mask, nasal cannula, or a tube inserted directly into the airway. The objective is to sustain oxygen saturation between 85% and 90%, aiming to decrease the fraction of inspired oxygen (FiO_2_) to below 65% within the initial 24-hour period.	The most common treatment for ARDS. Special care must be taken to avoid hyperoxia since it has been associated with increased proinflammatory responses and increased mortality (6).
**Mechanical ventilation**	Required by most ARDS patients to facilitate oxygenation. It seeks to sustain sufficient oxygenation and carbon dioxide elimination, while minimizing further lung damage and facilitating lung recovery.	Lower tidal volume (6ml/kg of predicted body weight) reduced the mortality rate in ARDS patients (7).
**Positive End-expiratory Pressure**	It aims to improve hypoxemia and atelectasis. Optimal PEEP levels must be patient-tailored, preventing lung overdistention and potential cardiovascular impact, requiring meticulous adjustment.	The survival benefit is controversial, but higher PEEP might lead to better outcomes (8).
**Prone positioning**	For patients with moderate to severe ARDS. Achievement of an even distribution of gravitational force in the tissue during mechanical ventilation.	Recommended for at least 12 hours per day to improve oxygenation (9). Prone positioning is believed to mitigate Ventilator-Induced Lung Injury (VILI) by engaging lung areas at the bottom and decreasing overinflation in upper regions (10).
**Extra-corporeal membrane oxygenation (ECMO)**	This is a more advanced treatment option for severe cases of ARDS, where a machine is used to oxygenate the blood outside the body. ECMO serves as supportive care for ARDS, not addressing its cause. It’s considered when conventional therapy fails oxygenation or CO_2_ removal, bearing possible complications.	Recommended as an intervention of last resort for patients who continue to experience hypoxemia despite the application of established conventional oxygenation treatments (5).
**Conservative fluid management**	Conservative fluid management in ARDS aims to limit pulmonary edema, enhance oxygenation, support hemodynamics, increase ventilator-free days, and prevent organ dysfunction by minimizing fluid accumulation. Special care should be taken to avoid hypovolemia.	Restrictive fluid management is suggested for all patients. Nevertheless, no change in the mortality rate was found in a fluid management trial (11).

Hypoxia is a hallmark of ARDS and is known to play a central role in the pathophysiology of the disease. Hypoxia can initially be an inflammatory stimulus that promotes proinflammatory responses and disrupts tissue barriers (13). The molecular mechanism involves the activation of NF-κB, a transcription factor that mediates the expression of proinflammatory genes (14). However, stabilizing hypoxia-inducible factors (HIFs), a group of transcription factors responsible for regulating the expression of numerous genes that promote cellular adaptation and survival, triggers anti-inflammatory responses to provide endogenous protection. Notably, the extracellular adenosine pathway, augmented during ARDS, -as evidenced by the upregulation of enzymes involved in adenosine production and the downregulation of genes that act in the termination of the adenosine signaling pathway-, is driven by HIFs and results in mitigating excessive inflammatory responses and reinforcing barrier function during ARDS (15–18). This highlights the importance of studying the hypoxia-adenosine axis during ARDS to optimize treatment strategies and improve patient outcomes. This review delves into the sophisticated relationship between hypoxia and adenosine signaling, encompassing adenosine generation, receptors, and metabolism. We will also address the upregulation and stabilization of hypoxia-inducible factors in ARDS. Finally, we will examine the diverse preclinical studies and current trials for clinical strategies to treat acute lung injury through therapeutic targeting of the hypoxia-adenosine axis.

## Hypoxia signaling during ARDS

### HIFs - PHD system

Reduced oxygen availability in tissues or organs plays a significant role in various human diseases (19, 20), including inflammatory conditions such as ARDS. HIF is stabilized during hypoxia via various mechanisms (21, 22). There are two primary components of HIF: the alpha subunits (HIFα), encompassing the oxygen-dependent HIF-1α, HIF-2α, and HIF-3α, and the constitutive beta subunit (HIF1β). The stability and activity of HIFs are tightly regulated by a family of oxygen-sensing enzymes called prolyl hydroxylase domain (PHDs). Under normoxic conditions (normal oxygen levels), PHDs hydroxylate specific proline residues on the HIFα subunit. This modification allows the von Hippel-Lindau tumor suppressor protein (pVHL) to recognize and bind to the HIFα proteins. As a result, the HIFα proteins are labeled for degradation by the proteasome (23). Conversely, PHD activity is inhibited under hypoxic conditions, allowing HIFα to accumulate and translocate into the nucleus, which binds to the HIF1β subunit. This complex binds to hypoxia-responsive elements (HRE) within the promoter region of target genes, activates their transcription, and enables cellular adaptation and survival during hypoxia (24). Three PHD isoforms exist, namely, PHD1, PHD2, and PHD3, each with distinct roles regulating HIFs (25). All three isoforms are sensitive to oxygen concentrations; however, the oxygen-dependent inhibition pattern differs among them. When the oxygen concentrations fall below 120 μmol/L (65 mmHg), which is lower than physiological levels for most tissues and would be considered hypoxic, PHD1, PHD2, and PHD3 are inhibited (26). In ARDS, the oxygen concentration can drop below 60 mmHg and in the lungs the induction of hypoxia-triggered genes occur at approximately 50–60 mmHg (27). PHD1 regulates HIF-2α-mediated transcription and has been linked to cellular metabolism, proliferation, and apoptosis (28). PHD2 is the main oxygen sensor and plays a crucial role in regulating HIF-1α under normoxic conditions, while PHD3 is the most relevant HIF-1α-regulating isoform in hypoxia (25, 29). The PHD isoforms have different expression patterns, tissue distribution, subcellular localization, and abilities to hydroxylate HIF-α subunits (26).

### HIFs during ARDS

HIFs and PHDs play critical roles in various physiological and pathophysiological processes in the lung. In ARDS, hypoxia signaling activates adenosine signaling to inhibit the inflammatory response in the lungs (30). Some examples of HIFs target genes in this pathway, which harbor HRE in their promoter, are the adenosine-producing enzyme CD73, the adenosine transporters ENT1 and ENT2, and the adenosine receptors A2AAR and A2BAR. Moreover, HIFs can upregulate in response to hypoxia the expression of genes involved in angiogenesis (such as VEGF and NOS, among others) (31), leading to the development of new blood vessels in the lung. This can help to increase oxygen delivery to hypoxic tissues and promote tissue repair. Furthermore, HIFs can regulate gene expression in the immune response, including cytokines, chemokines, and adhesion molecules (32). This can help to recruit immune cells to the site of injury or infection in the lung, allowing them to clear invading pathogens and promote tissue repair. Inhibition of PHDs is beneficial in animal models of acute lung injury, likely due to the activation of HIFs and the subsequent upregulation of genes involved in tissue repair and anti-inflammatory signaling (33). Another study demonstrated that enhancing glycolysis by PHD inhibition protected alveolar epithelial cells from acute lung injury (34). Additionally, the HIF PHD inhibitor Roxadustat was observed to stabilize HIF-1α and have regenerative potential in a mouse model of hyperoxia-induced lung injury (35). Moreover, in endotoxin-mediated injury, the endothelial adherens junction integrity improved due to the enhancement of vascular endothelial protein tyrosine phosphatase (VE-PTP) via HIF-2α activation (36). These studies point out the potential therapeutic benefits of enhancing HIF in the context of acute lung injury.

Changes in cellular metabolism, such as modifications in glycolysis and fatty acid oxidation (FAO), have become crucial mechanisms in various pathological conditions, including inflammation (37). Recent research presents compelling evidence that carbohydrate metabolites and intermediates, beyond their traditional role as an energy source, have significant functions in controlling inflammatory processes and the potential to alleviate lung inflammation (38). HIF-1α stabilization was shown to play a protective role by regulating glucose metabolism in alveolar epithelial cells, ultimately reducing lung inflammation and pulmonary edema (38). Mice with alveolar epithelial cell-specific *Hif1a* deletion showed increased lung inflammation. Succinate, a tricarboxylic acid cycle intermediate, has been shown to have a protective role in alveolar epithelial cells during ARDS by enhancing HIF-1α levels (39). The succinate-mediated HIF stabilization takes place through distinct mechanisms. Firstly, succinate induces the production of Reactive Oxygen Species (ROS) by inhibiting succinate dehydrogenase (SDH), consequently stabilizing HIF. This stabilization occurs via ROS-mediated oxidation of Fe^2+^, an essential cofactor for PHD activity, thus limiting its function (40). Additionally, succinate can traverse from mitochondria to the cytosol through specific transporters. Excessive cytosolic succinate impedes PHD activity, resulting in HIF stabilization and activation, an occurrence called ‘pseudohypoxia’ (41). In an *in vitro* setting, the addition of succinate reduced epithelial inflammation following stretch. In a mouse model featuring inducible alveolar-epithelial *Sdha* deletion (*Sdha^loxp/loxp^*/SPC-CreER mice), several favorable outcomes were observed, including diminished lung inflammation, improved integrity of the alveolar barrier and mitigated histological changes towards injury. This aligns with the notion that succinate plays a functional role in stabilizing HIF. Notably, *Sdha^loxp/loxp^*/SPC-CreER mice exhibited heightened HIF-1α levels in response to hypoxia or acute lung injury (ALI) (39). These findings suggest that targeting succinate and its related pathways may have potential therapeutic benefits in ARDS.

Sepsis is one of the causes leading to ARDS. Thiel et al. investigated the role of HIF-1α in T cells under hypoxic conditions in the context of sepsis and found that deleting the HIF-1α gene in T cells improved survival in septic mice (42). This was attributed to preventing T-cell inhibition in hypoxic inflamed tissues. Moreover, T lymphocyte development and activity have been shown to be influenced by hypoxia (43). For instance, cytotoxic T lymphocytes (CTLs) development is delayed at 2.5% oxygen compared to 20%. However, the development of CTLs at 2.5% oxygen is more sustained, and the CTLs are much more lytic. The results indicate that targeting HIF-1α in T cells could be a therapeutic strategy for improving survival in septic conditions.

## HIF/adenosine axis during ARDS

### Adenosine biology

**Figure 2:**
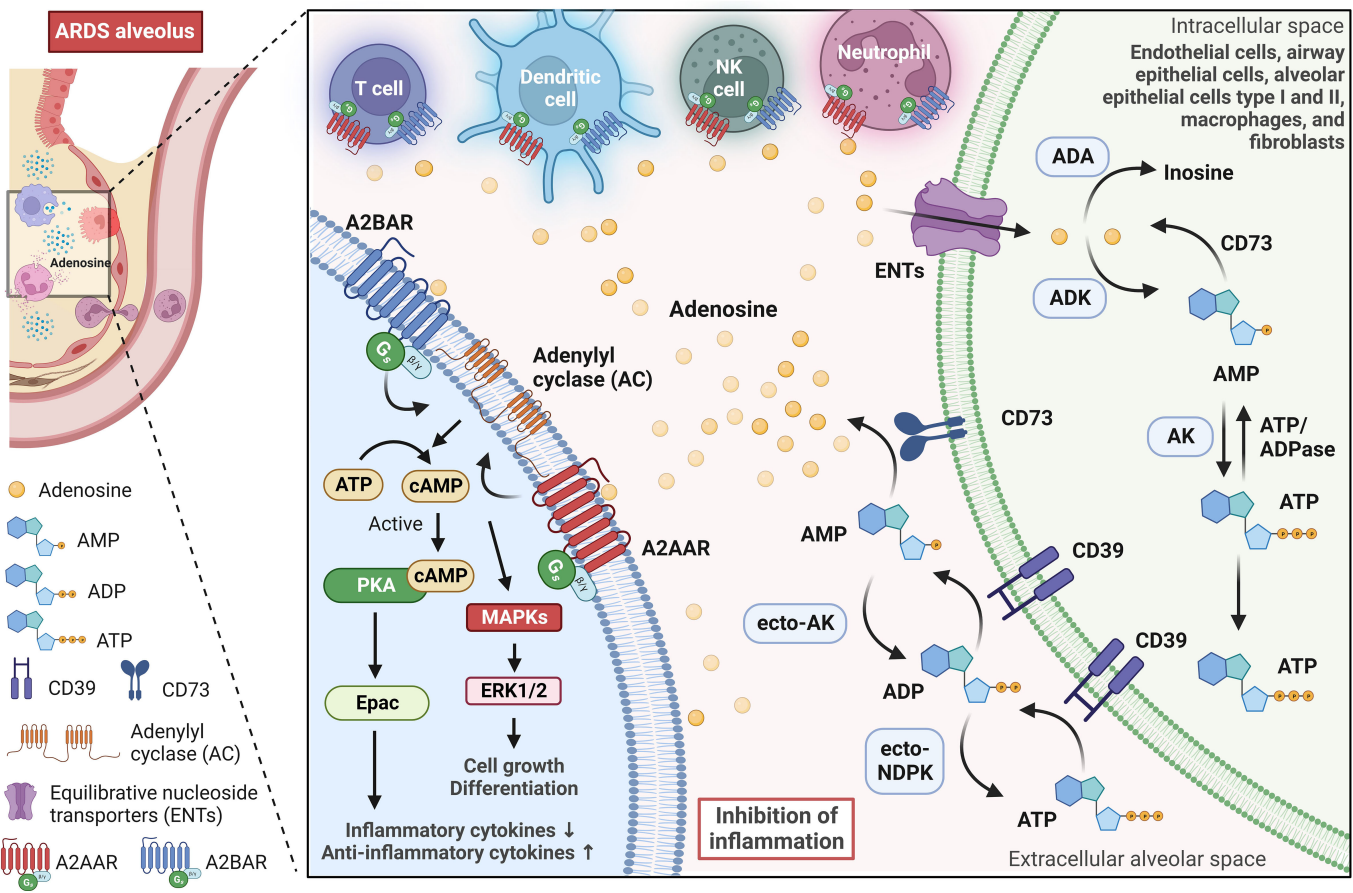
Adenosine signaling pathway in the alveolar microenvironment during ARDS. Adenosine is synthesized on the surface of cells, especially during stressful situations when ATP is released into the extracellular alveolar space. In the lungs, adenosine-producing cells (green cell on the right) involve endothelial cells, airway epithelial cells, alveolar epithelial cells type I and II, macrophages, and fibroblasts. The extracellular ATP is broken down by ectonucleotidases CD39 and CD73, which leads to the production of extracellular ADP, AMP, and finally, adenosine. Further regulation of extracellular AMP and ADP levels is carried out by enzymes such as adenylate kinase (ecto-AK) and nucleoside diphosphate kinase (ecto-NDPK), which convert AMP back to extracellular ATP. In the alveolar environment, extracellular adenosine primarily activates G-protein-coupled adenosine receptors A2AAR and A2BAR. These receptors are expressed in alveolar epithelial cells and various immune and inflammatory cells, and they play a significant role in modulating immune responses. A2AAR and A2BAR signal via a Gs protein and activate adenylyl cyclase, leading to an increase in intracellular cyclic adenosine monophosphate (cAMP), which in turn, activates protein kinase A (PKA) and the exchange protein directly activated by cAMP (Epac). PKA and Epac pathways suppress inflammatory cytokine production while strongly elevating anti-inflammatory cytokines. Furthermore, A2AAR and A2BAR can also influence the activation of mitogen-activated protein kinases (MAPKs), such as ERK1/2, which are involved in various cellular activities, including cell growth and differentiation. Equilibrative nucleoside transporters (primarily ENT2) regulate extracellular adenosine levels in the lung by mediating its uptake from the extracellular space into cells. Intracellular adenosine is metabolized into inosine by adenosine deaminase (ADA) or AMP by adenosine kinase (ADK). This image was created using BioRender.com.

Adenosine is a naturally occurring molecule in the body that plays a significant role in various physiological processes. It is produced in all cells of the body, mainly by the breakdown of adenosine triphosphate (ATP) and other sources like NAD^+^ or through hydrolysis of S-adenosyl homocysteine (44, 45). Adenosine synthesis can occur inside or on the cell surface (46). The latter has been associated with cellular stress and the release of ATP from inside cells into the extracellular space, either through leakage or controlled release pathways involving connexin and pannexin channels or ATP-binding cassette transporters (47, 48). In the ARDS scenario, extracellular adenosine produced mainly by endothelial cells, alveolar epithelial cells, alveolar macrophages, and neutrophils provides protective effects (49). In the extracellular space, the classical pathway for generating adenosine consists of the breakdown of extracellular ATP through the action of ectonucleotidases, such as CD39 (also known as ectonucleoside triphosphate diphosphohydrolase 1) and CD73 (also known as 5′-nucleotidase) (**Figure 2**). Ectonucleotidases are tethered to the plasma membrane and possess their active sites facing the extracellular milieu, facilitating the enzymatic breakdown of nucleotides present outside the cell (50). This spatial orientation enables these enzymes to participate in the modulation of extracellular nucleotide concentrations, thereby influencing purinergic signaling pathways. CD39 is an enzyme found on cell membranes, and it catalyzes the hydrolysis of extracellular ATP, resulting in the production of extracellular ADP and AMP. Then, extracellular AMP is dephosphorylated into adenosine by CD73, an enzyme anchored by glycophosphatidylinositol, and can also exist in a soluble form (51). Extracellular AMP levels are additionally modulated by secreted or membrane-associated enzymes such as ecto-adenylate kinase (ecto-AK) and ecto-nucleoside diphosphate kinase (ecto-NDPK) (52). These enzymes regulate the conversion of AMP back to extracellular ATP.

Extracellular adenosine has a short half-life and exerts tissue function by stimulating four G-protein-coupled adenosine receptors: A1AR, A2AAR, A2BAR, and A3AR (53). Adenosine receptors are conservative among vertebrate species (54). Mice engineered with targeted deletions of each of these receptors exist and have played a pivotal role in elucidating the physiological and pathological functions associated with these receptors. Under normal physiological conditions, adenosine concentrations are adequate to activate A1AR, A2AAR, and A3AR, mainly if these receptors are abundantly expressed (46). In contrast, A2BAR requires higher concentrations of adenosine to achieve significant activation, concentrations that are typically found in more extreme or pathological situations. The adenosinergic pathway plays an essential role in modulating immune and inflammatory responses in the body (55). Adenosine can act as an immunosuppressive metabolite, mainly activating A2AAR and A2BAR. This activation initiates the cAMP/PKA/CREB pathway, which is instrumental in suppressing immune cells and helping tumors evade the immune system (56). Moreover, adenosine inhibits the release of anti-tumor cytokines by phosphorylating CREB in T cells (57). Adenosine also has a significant impact on regulatory T cells (Tregs), as it increases their population and enhances their immune regulatory functions (58–60). Adenosine can also affect dendritic cells (DCs), stimulating a more tolerogenic phenotype (53).An autocrine action of adenosine has been reported in T regulatory cells (Tregs) via CD73 and A2AAR, which is fundamental for their ability to suppress innate immune responses (48). In models of kidney ischemia-reperfusion injury, the absence of CD73 or A2AAR in Tregs renders them ineffective in preventing injury (61). Moreover, adenosine’s autocrine loops have also been demonstrated in neutrophils, modulating migration towards chemoattractants (62), and in tumor environments, contributing to immunosuppression in CD73^+^CD4^+^ effector T cells (63). Extracellular adenosine signaling is regulated by equilibrative nucleoside transporters (ENT1 and ENT2) that mediate adenosine uptake from the extracellular milieu to the intracellular room. ENT1, widely present in human and rodent tissues, predominantly regulates adenosine signaling during hypoxia (64). In contrast, ENT2 is ubiquitous but highly expressed in tissues like vascular endothelium and skeletal muscle; it has lower adenosine affinity but transports various nucleosides (65). Adenosine is then metabolized intracellularly into inosine by adenosine deaminase (ADA) or AMP by adenosine kinase (ADK). In immune function, adenosine plays a central modulatory role in inflammatory responses in both physiological and pathological conditions.

### Cross-talk between hypoxia and adenosine signaling during ARDS

Over the past decade, investigators have identified multiple genes under the regulatory influence of HIFs, which govern the modulation of extracellular adenosine metabolism and signaling (66). HIF-driven adenosine metabolism and signaling play a pivotal role in safeguarding tissues during conditions such as myocardial injury (67), inflammatory bowel disease (68), and acute lung injury (69). Both HIF-1α and HIF-2α are involved in adenosine pathway, and many of their targets overlap since both recognize the same consensus DNA-binding element in their target gene promoter region (70). Thus, their functional role is given by their tissue/cell-specific expression profile (71). For instance, HIF-1α has been shown to orchestrate adenosine efflux in hepatocellular carcinoma (72), while HIF-2α has been found to promote angiogenesis through A2AAR signaling in pulmonary endothelial cells (71). Moreover, HIFs further influence gene expression in adenosine-related pathways. ENT1 and ENT2 promoters, along with CD73, A2AAR, and A2BAR, contain hypoxia response elements (HREs), making them transcriptional targets of HIFs. During ARDS, hypoxia signaling prominently affects lung endothelial cells (LECs), alveolar epithelial cells (AECs), infiltrating leukocytes, and monocytes. LECs and AECs, lining blood vessels and alveoli, respectively, are pivotal in regulating inflammatory lung injury and repair (73, 74). Infiltrating leukocytes and monocytes also undergo hypoxia-driven changes, influencing the inflammatory response within the lungs (75). HIFs 1 and 2 orchestrate these cellular responses, modulating a spectrum of target genes that regulate critical processes, including cell survival, differentiation, migration, and proliferation (75, 76).

#### Extracellular adenosine generation

Inflammation or hypoxia triggers in various cell types the release of adenosine precursor molecules, mainly ATP and ADP, into the extracellular space through mechanisms such as vesicular exocytosis, opening of conductive pathways or through the action of connexins and pannexins (77–79), but also due to the cell membrane damage (80). ATP can activate proinflammatory responses by binding to the purinergic ionotropic P2X7 receptor and eliciting NLRP3 inflammasome activation (81). Hypoxia-induced enzymatic processes convert ATP and ADP into adenosine, shifting the signaling balance from proinflammatory to anti-inflammatory. Research examining the impact of hypoxia on gene transcription has unveiled that under such circumstances, CD39 undergoes an increase in transcription with dependence on an SP1-mediated pathway (82). Likewise, CD73 transcriptional induction occurs via HIF-1α binding to the CD73 promoter, resulting in elevated CD73 transcript and protein levels (83, 84). Interestingly, Sp1 and HIF-1α transcription factors regulate their activities reciprocally (85). Sp1 induces HIF1A gene transcription, while HIF-1α binds to SP1 gene promoter, upregulating Sp1 expression and influencing cellular adaptation to low oxygen levels (86). In regulatory T cells, which, among others, are responsible for regulating excessive inflammatory responses, an upregulation of CD73 expression driven by HIF-1α stabilization was demonstrated to potentiate their immunosuppressive effects via adenosine on effector T cells (87, 88). Deletion of CD39 and CD73 was shown to exacerbate acute lung injury in mechanically ventilated mice (89). Treatment with soluble CD39 and CD73 compounds showed promise in attenuating mechanical ventilation-induced lung injury and improving survival, suggesting a therapeutic strategy for non-infectious lung pathologies.

It is important to note that in cancer, the concentration of adenosine is elevated in the tumor microenvironment (TME) due to the high expression of the adenosine-generating ectoenzyme CD73 by tumor cells. This can lead to the suppression of immune responses against the tumor. By supplementing oxygenation, the hypoxic conditions in the TME can be reduced, which in turn improves the efficacy of anti-PD-1 therapy (90). These findings suggest that supplemental oxygenation could be a potential strategy to enhance the effectiveness of immunotherapies for cancer treatment.

#### Adenosine receptors

Adenosine receptors are expressed in various cell types throughout the body, mainly but not limited to epithelial, endothelial, nerve, muscle, and immune cells, including neutrophils and macrophages. Factors like hypoxia, cytokines, and neuronal activity regulate their expression across various cell types (46, 91). Comparative studies revealed selective HIF-1α dependent induction of A2BAR in various tissues, including the lung, during inflammation or hypoxia (92). A2BAR is a crucial connection between hypoxia and adenosine signaling in cases of acute lung damage. In a normobaric hypoxia (8% O_2_, 92% N_2_) mouse model, Eckle et al. revealed that *A2bar^-/^*^-^ mice had significantly higher vascular permeability in comparison to *A1ar^-/-^*, *A2aar^-/-^*, or *A3ar^-/-^* mice (93). This indicates that A2BAR plays a specific role in endothelial leakage in response to hypoxia. Consequently, the A2BAR inhibitor PSB1115, caused significant pulmonary edema in hypoxia-exposed C57BL/6 mice, and the highly selective agonist BAY 60-6583, significantly attenuated hypoxia-induced pulmonary edema (93). The same results were obtained in ventilator-induced lung injury and endotoxin-induced lung injury (94, 95). In a study by Hoegl et al., the protective role of alveolar epithelial A2BAR in acute lung injury (ALI) was investigated (96). This study highlighted that the expression of A2BAR in alveolar epithelial cells contributes to mitigating ALI. Mice with specific *A2bar* deletion in alveolar epithelial cells exhibited detrimental effects similar to those observed in whole-body A2BAR knockout mice during ALI. Aerosol treatment with BAY 60-6583 in a two-hit model (LPS + VILI) was found to improve lung injury and alveolar fluid clearance, emphasizing the protective role of A2BAR expressed by alveolar epithelial cells in ALI. This targeted approach for delivering compounds directly to alveolar epithelial cells through inhalation holds promise for minimizing potential systemic side effects. These findings reveal a role for A2BAR signaling in the alveolar-capillary barrier during ARDS and implicate A2BAR agonist as a potential therapeutic target.

A2AAR is also transcriptionally induced in pulmonary endothelial cells during hypoxia through HIF-2α-controlled pathways (71). Its activation has been extensively studied for its potent anti-inflammatory properties. In a groundbreaking study, Ohta and Sitkovsky discovered that A2AAR is crucial in reducing inflammation and preventing tissue damage *in vivo (* 97). The investigation uncovered a mechanism of hypoxia-driven adenosine-mediated immunosuppression, whereby an increase in the level of extracellular adenosine triggers intracellular signaling pathways that result in the downregulation of inflammation and protection from tissue damage through the activation of A2AAR. Research conducted by Reece et al. demonstrated that A2AAR activation during early reperfusion in a pig lung transplantation model reduced lung inflammation and preserved lung function, suggesting its therapeutic potential (98). A specific A2AAR agonist, ATL202, was found effective in reducing immune cell recruitment and cytokine production in a mouse model of acute lung injury (ALI) (99). Notably, A2AAR on myeloid cells appeared to play a role in controlling immune cell recruitment. Adenosine-mediated activation of A2AAR suppresses the secretion of the pro-inflammatory molecule TNF-α in macrophages (100, 101). A recent study has shown that vessel-associated macrophages initiate PMN migration during inflammation by stimulating endothelial cells to form ICAM-1 “hot spots” that support PMN transendothelial migration. Macrophage-derived TNF-α activates the endothelial TNFR2 axis, which is essential for this mechanism (102). Therefore, activating A2AAR by adenosine reduces TNF-α levels, thus suppressing immune cell recruitment. Additionally, the activation of peroxisome proliferator-activated receptor-γ (PPARγ) was shown to reduce lung injury in an A2AAR-dependent manner, and the combined application of PPARγ and A2AAR agonists exhibited a synergistic effect in suppressing inflammation and improving lung function (103). Post-treatment with CGS21680, an A2AAR agonist, also reduced neutrophil infiltration and lung tissue damage in a mouse model of pleurisy (104). These findings collectively suggest that A2AAR agonists hold promise for treating various inflammatory lung diseases.

#### Termination of adenosine signaling (uptake and metabolism)

The termination of adenosine signaling is also regulated by hypoxia. Studies utilizing radiolabeled adenosine demonstrate a decelerated extracellular adenosine uptake in hypoxic conditions. This was coupled with a notable reduction in the expression of ENT1 and ENT2, prominent adenosine transporters within vascular endothelial and epithelial cells (105, 106). HIF-1α plays a pivotal role in modulating the expression of ENT1 and ENT2, and the hypoxia-induced downregulation of these transporters mitigates inflammatory responses. Consequently, the absence of HIF-1α in epithelial cells leads to an elevation in ENT expression. Studies revealed that in the lung, mainly ENT2 mediates the termination of the adenosine signaling and that inhibition of ENT2 results in lung protection via A2BAR activation (107, 108). Similar results were observed in intestinal inflammation, where repression or deletion of epithelial ENT2 favors the resolution of inflammation through A2BAR signaling (109). Interestingly, ENT1 was shown to be associated with adenosine termination in myocytes and erythrocytes and ENT1 inhibition with cardioprotection and faster acclimatization to high altitudes, respectively (110, 111). Furthermore, hypoxia exerts inhibitory effects on adenosine kinase (AK) through HIF-1α-mediated transcriptional repression (112). This inhibition results in an augmented release of extracellular adenosine and a consequential reduction in vascular leakage during hypoxic conditions. Mechanistically, HIFs can act indirectly through additional molecules that may exert repressive effects on members of the adenosine signaling pathway. Such is the case of microRNAs (miRNAs), which are short non-coding RNAs involved in gene expression through post-transcriptional processes (113–115). Nowadays, hypoxia-regulated miRNAs, collectively known as hypoxamirs, are recognized as pivotal components in the cellular response to hypoxia (116). Some examples of miRNAs upregulated by hypoxia that target members of the adenosine signaling pathway are miR-30b (targets CD73) (117), miR-146b (targets ADA) (118), miR-155 (targets HIF-1α itself, proposed as negative-feedback loop during prolonged hypoxia) (119). Altogether, hypoxia orchestrates adenosine synthesis, signaling, and termination via hypoxia-dependent induction of genes constrained in the adenosine signaling pathway.

### Alternative HIF targets that enhance adenosine signaling

Under hypoxic conditions, various HIF dependent molecular signals can modulate the adenosine signaling pathway. An example of such a signal is netrin-1 (NTN1), classified as a member of neuronal guidance proteins involved in axon guidance and cell migration. NTN1 is expressed in multiple tissues, including the heart muscle, brain, adrenal gland, kidney, liver, lung, and others. At the cellular level, besides glial cells that display the highest levels, it is expressed in cardiomyocytes, epithelial, endothelial, and germ cells (available from proteinatlas.org). Recent studies also demonstrated its anti-inflammatory properties in atherosclerotic cardiovascular disease, inflammatory bowel disease, and acute lung injury (120–123). Hypoxia increases extracellular adenosine levels and elevates NTN1 levels through direct transcriptional induction of its gene expression (124). Intriguingly, NTN1 appears to potentiate signaling through the adenosine receptor A2BAR, inhibiting leukocyte migration and serving as a robust limiting factor during situations of oxygen scarcity and inflammation (55, 125). By binding to A2BAR, NTN1 increases intracellular cAMP levels, which mediates its effects (124). The precise mechanism underlying netrin-1’s augmentation of extracellular adenosine signaling remains elusive. Nonetheless, we can speculate that this interaction may represent a mechanism through which A2BAR is stimulated, even if adenosine levels decrease below the concentration required to activate A2BAR. This could, therefore, help prevent tissue injury following episodes of hypoxia and ischemia, and assist inflamed tissue in returning to its usual state of balance.

## Therapeutic targeting of the HIF/adenosine axis for ARDS

**Figure 3:**
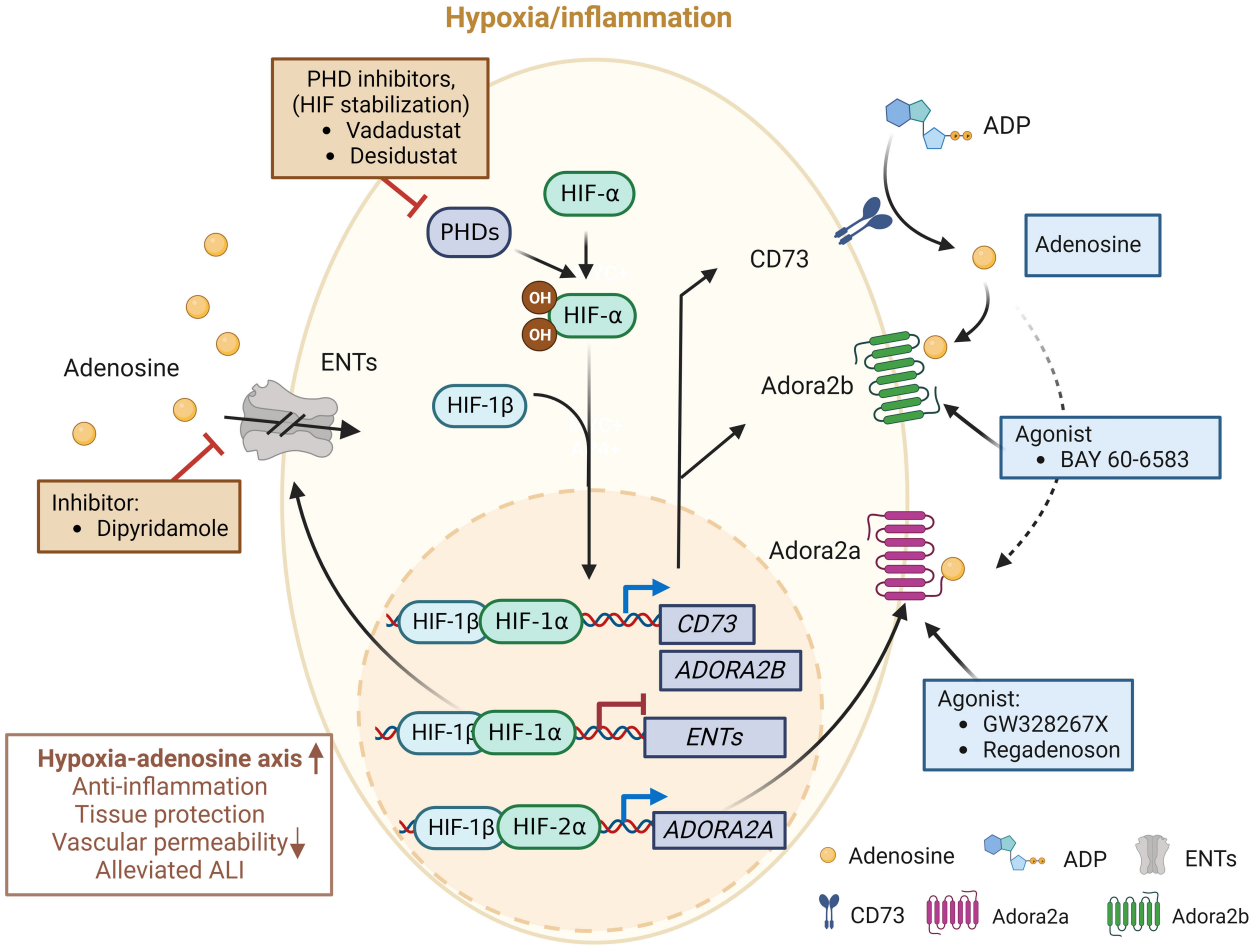
Molecules in clinical trials aimed at modulating the hypoxia-adenosine axis. Currently, interventions are being evaluated to regulate inflammatory responses and develop treatments for various lung diseases, including ARDS, by targeting different stages of the adenosine signaling pathway. Molecules in blue boxes represent stimulators, while molecules in red boxes are inhibitors. This image was created using BioRender.com.

The balance between oxygen supply and demand is essential during physiological cellular activities. As previously referred, hypoxia stabilizes HIFs and affects the expression of many genes that are significant in ARDS (75). Studies have provided compelling evidence that HIF and adenosine signaling are tightly linked to modulating inflammatory responses during lung injury (69, 71, 126, 127). For this reason, researchers have made significant efforts to intervene in the hypoxia-adenosine axis to develop targeted treatments for ARDS (**Figure 3**). In this section, we summarize ongoing clinical trials targeting the hypoxia-adenosine axis to treat ARDS and other lung-related diseases (**Table 2**).

**Table 2:** Therapeutic approaches for lung disease targeting the hypoxia-adenosine axis.

Target	Drug	NCT Number	Study Status	Conditions	Phase
**Hypoxia-inducible factor-prolyl hydroxylase (PHD) inhibitor**	Vadadustat; AKB-6548 or Placebo	NCT04478071	Completed	Acute Respiratory Distress Syndrome | Coronavirus Infection	Phase II
	Desidustat	NCT04463602	Completed	COVID-19 | Lung Inflammation	Phase II
**A1AR antagonist**	PBF-680 or Placebo	NCT05262218	Recruiting	Obstructive Pulmonary Disease	Phase II
**A2AAR agonist**	GW328267X or Saline	NCT01640990	Completed	Acute Lung Injury	Phase I
	Regadenoson or Dobutamine	NCT00763035	Terminated	Coronary Artery Disease | Asthma | Chronic Obstructive Pulmonary Disease | Angina	Early phase I
	Regadenoson or Placebo	NCT00862641	Completed	Asthma | Coronary Artery Disease | Chronic Obstructive Pulmonary Disease	Phase IV
	Regadenoson	NCT04606069	Recruiting	COVID-19 | Lung Inflammation	Phase I Phase II
**Ecto-nucleotidase activators/inhibitors**	AK119 (humanized monoclonal antibody against CD73)	NCT04516564	Completed	COVID-19	Phase I
**Adenosine availabilit**y	Aerosolized inhaled Adenosine	NCT04588441	Not yet recruiting	COVID-19	Phase II

### Clinical application of HIF-PHD inhibitors

HIF-PHD inhibitors (HIF-PHIs) are an encouraging new category of drugs that can impact the adenosine signaling pathway. These molecules are designed to inhibit HIF-PHDs, stabilizing HIFs under normoxic conditions (128). Among these inhibitors, Daprodustat (GSK-1278863) is administered orally and was the first to receive approval for treating anemia in patients with dialysis-dependent chronic kidney disease (CKD) by the Food and Drug Administration in the US. Other HIF-PHIs, including Roxadustat (FG-4592), Vadadustat (AKB-6548), Molidustat (BAY 85-3934), Enarodustat (JTZ-951), and Desidustat (ZYAN1), have already completed or are presently engaged in various clinical trials for their potential future applications (129). Several HIF-PHIs are approved for clinical usage in Japan and the European Union (129). Recently, HIF-PHIs have been recognized as potential novel treatment approaches for clinical trials involving COVID-19 patients (130). For example, Vadadustat (NCT04478071) and Desidustat (NCT04463602) are currently being evaluated in a phase II clinical trial with the expectation of preventing the onset of ARDS in COVID-19 patients who are hospitalized (131, 132). The pharmacological HIF activators could potentially be effective in enhancing HIF-driven extracellular adenosine signaling in COVID-19 patients, which may help provide tissue protection during adenosine-dependent immunosuppression (133, 134). Finally, conservative oxygen therapy could also serve as an approach to promote HIF stabilization during ARDS (135). However, the optimal oxygenation target in ARDS patients for best clinical outcomes will need to be determined in future clinical studies.

Evidence has shown that oxygenation can exacerbate inflammatory reactions, contributing to tissue damage and the development of acute respiratory distress (ARDS) (136). This response has been linked to reduced levels of endogenous adenosine, which usually activates A2AAR on immune-competent cells, mainly pulmonary invariant natural killer T (iNKT) cells, upon injury (137). Interestingly, similar effects could be partially replicated by using an A2AAR agonist. As a result, a proposal emerged suggesting that oxygen therapy should be administered combined with an A2AAR agonist (136). This idea aligns with the notion that A2AAR agonists could be valuable in preventing various forms of ischemia-reperfusion damage, a concept supported by substantial experimental evidence (138).

### Clinical modulation of adenosine receptors

A2AAR has been extensively researched in recent decades because of its potential for different therapeutic uses. In particular, the expression of A2AAR is widespread in the lungs, including on bronchial epithelial cells and immune cells (139). In this regard, selective A2AAR agonists have been suggested to have a significant impact on airway inflammation and injury to lung tissue (140). Among the A2AAR agonists, GW328267X is a potent and selective agonist for A2AAR and a competitive antagonist at the adenosine A(3) receptor (141). However, two subsequent clinical trials were discontinued due to poor efficacy against allergic rhinitis (intranasal) (142) and asthma (inhaled) (143). The A2AAR agonist Regadenoson, among vasodilatory agents, is recognized for its effectiveness in promoting near-maximal coronary hyperemia without inducing bronchospasm in patients with coronary artery disease who have advanced lung disease (144). Certainly, over the past decade, there has been an increasing amount of evidence that supports the safety of regadenoson for patients with asthma and COPD, showing no variations in lung function parameters (145–147). Furthermore, following the outbreak of COVID-19, it was observed that a cytokine storm, a phenomenon where an excessive release of cytokines occurs due to an overreaction to the virus, could potentially be the primary cause of respiratory failure, which is seemingly the leading cause of death among severe COVID-19 patients (148). Hence, investigations conjectured that regulating the occurrence of the cytokine storm may assist in alleviating symptoms and aiding recovery in severe COVID-19 patients. A Phase I/II clinical trial enrolled participants to examine the hypothesis that regadenoson facilitates clinical improvement and enhances survival rates in COVID-19 patients when compared to patients who are undergoing a placebo-control (NCT04606069), the trial was recently completed but results are not available yet. Conversely, pentoxifylline, an A2AAR antagonist (149), has also shown anti-inflammatory effects (149–152), showcasing the complexity of therapeutic targeting of A2AAR in inflammatory diseases. Despite encouraging experimental data, the absence of A2BAR agonist testing in patients, particularly compounds like BAY-60-6583, remains a missed opportunity, with reasons for exclusion from clinical ARDS trials unclear.

Preclinical data indicates that extracellular CD73‐mediated adenosine production exhibits a protective role in non-infectious lung pathologies. However, it has been hypothesized that during SARS-CoV-2 infections, inhibition of CD73 may increase extracellular ATP, which may raise the production of IFN-β and trigger antiviral responses. A first-in-human (FIH), phase I, placebo-controlled, single ascending dose study to evaluate the safety, tolerability, pharmacokinetics, and immunogenicity of AK119, a humanized monoclonal antibody targeting the CD73, was conducted with the future aim of treating COVID-19 patients (NCT04516564) (153). Interestingly, alterations in the CD39/CD73 axis of T cells have been associated with SARS-CoV-2-induced ARDS. For example, higher frequencies of CD39^+^ memory T effector cells and lower frequencies of CD73^+^ T reg cells were found in severe compared to mild COVID-19 patients (154, 155), while its functional contribution remains unclear. Taken together, therapeutic targeting of CD73 for ARDS is still in its infancy.

Initial studies on the potential therapeutic effects of inhaled adenosine in patients in Italy and the US with severe COVID-19 demonstrated a significant improvement in oxygenation, reduced inflammation, and improved clinical outcomes in most treated patients (156, 157). While the study provides promising initial findings, it is based on fewer cases. An upcoming clinical trial, the ARCTIC trial, will use aerosolized inhaled adenosine treatment in patients with ARDS caused by COVID-19 (NCT04588441). This phase II study aims to evaluate adenosine’s effectiveness in mitigating the excessive lung inflammation observed in COVID-19, as evidenced by improvements in lung function, resolution of clinically significant lung function indicators, and reduction in systemic inflammation and coagulation markers. The results from this trial will be instrumental in determining the functional role of extracellular adenosine during COVID-19 ARDS.

## Conclusion and challenges

The regulation of immune cell function in hypoxic and normoxic conditions is crucial, and hypoxia-inducible factors (HIFs) play a significant role in this process. HIFs are transcription factors that assist immune cells in adapting to hypoxic environments, such as infected or inflamed tissues. HIF-α stabilization can also occur during immunity and inflammation under normoxia, regulating metabolism and directly affecting immune genes. HIFs have been linked to numerous inflammatory diseases and immunosuppression in tumors. The differences in HIF-1α and HIF-2α functioning between cell types and environments are a challenging factor for therapeutic targeting. The connection between adenosine and signaling processes during hypoxic conditions is significant. Adenosine has the potential to impact oxygen availability and demand in diverse ways, both short-term and long-term, for instance, by promoting the development of new blood vessels (31). One intriguing aspect of adenosine’s function is its ability to regulate multiple facets of inflammation. It mitigates neutrophil-driven inflammation by curbing their recruitment, adhesion to endothelial cells, and reactive oxygen species production. It modulates cytokine production, notably inhibiting TNF-α and IL-12. Adenosine also suppresses the inflammatory activation of macrophages and fosters the recruitment of immature dendritic cells to sites of inflammation (the latter via A1AR and A3AR). Additionally, it influences regulatory T-cell function via A2AAR in hypoxic environments, contributing to the overall regulation of inflammation, especially within hypoxic conditions, triggering endothelial adenosine upregulation (53, 158, 159). Furthermore, the precise roles of adenosine receptors can vary depending on timing and the specific tissues involved. Their expression varies across tissues, leading to specific physiological responses. They have different affinities for adenosine, which modulate their responses under different conditions. These receptors also couple to distinct signal transduction pathways, further diversifying their impact. Prolonged exposure to agonists can trigger receptor desensitization, reducing its responsiveness (160). Adenosine receptor function is dynamic and context-dependent, and it is shaped by various factors such as hypoxia, ischemia, and circadian rhythms, elucidating their significance across different physiological and pathological states (161). Forthcoming studies in this matter should focus on aspects like a) exploring the correlation between hypoxia and adenosine, encompassing adenosine generation, its receptors, and processing, in acute and chronic lung ailments; b) investigating existing strategies to target the hypoxia-adenosine pathway therapeutically in various disease contexts; c) appraising the complexity of hypoxic-adenosinergic responses in the pathophysiology of ARDS and the development of lung fibrosis; d) studying the role of adenosine and HIFs in initiating vascular remodeling processes that contribute to the pathogenesis of ARDS; e) understanding cell-specific functions to develop targeted therapies for local lung delivery.

Results of investigations in the past decades highlight the fact that there is a reasonable rationale to evaluate the potential of adenosine-targeting therapies in ARDS. The large amount of ongoing clinical trials attests to this. A critical factor to consider when designing clinical trials is the clinical heterogeneity of ARDS. In about one-fifth of the patients that develop ARDS, the cause of lung injury is multifactorial and not due to a unique event. Moreover, mixed cases presenting direct and indirect components of lung injury can happen and should be analyzed carefully. Overall, the role of HIF in regulating inflammation and tissue repair in the lung is complex and context-dependent. Higher comprehension of the mechanisms governing hypoxia and adenosine signaling within the pulmonary context holds the potential to pave the way for innovative therapeutic approaches in the realm of ARDS.

Future research in ARDS should prioritize understanding its inherent heterogeneity to delineate distinct phenotypes based on sub-patient groups (e.g., septic ARDS, bacterial pneumonia, etc.) and facilitate targeted clinical trials (162). Optimizing established interventions including lung protective mechanical ventilation and prone positioning remains crucial (163). Establishing diagnostic protocols to identify treatable diseases within the ARDS diagnosis, evaluating the impact of secondary hits, and discerning patients with unresolved ARDS are pivotal (164). Given their higher mortality risk, it is imperative to explore novel therapeutic targets for older patients (165). Additionally, assessing the potential of therapeutic interventions developed for COVID-19 ARDS in other illness-induced ARDS and re-evaluating past research interventions, such as the prone position, are essential for refining ARDS management (166). Including different models in preclinical studies to verify findings will also be helpful. Collectively, these research avenues will enhance our comprehension of ARDS, optimize existing treatments, and unravel novel therapeutic avenues to improve patient outcomes.

## Author contributions

KF: Conceptualization, Investigation, Writing – original draft, Writing – review & editing. JK: Writing – original draft, Writing – review & editing. WR: Visualization, Writing – review & editing. TM: Writing – review & editing. HE: Conceptualization, Funding acquisition, Writing – original draft, Writing – review & editing. XY: Conceptualization, Funding acquisition, Writing – review & editing.
